# ST2-Conditioned Medium Fosters Dorsal Horn Cell Excitability and Synaptic Transmission in Cultured Mouse Spinal Cord

**DOI:** 10.1007/s12015-023-10618-x

**Published:** 2023-09-06

**Authors:** Esri H. Juárez, Chelsea R. Wood, Rebecca Davies, Oksana Kehoe, William E. B. Johnson, Adalberto Merighi, Francesco Ferrini

**Affiliations:** 1 Department of Veterinary Sciences, University of Turin, Largo Paolo Braccini 2, I-10095 Grugliasco, TO Italy; 2 Chester Medical School, University of Chester, Parkgate Road, Chester, CH1 4BJ UK; 3 School of Life Sciences, Coventry University, Coventry, CV1 2DS UK; 4 Centre for Regenerative Medicine Research, School of Medicine, Keele University, Keele, Staffordshire ST5 5BG UK; 5 Robert Jones and Agnes Hunt Orthopaedic Hospital, Oswestry, Shropshire SY10 7AG UK; 6 Department of Psychiatry and Neuroscience, Université Laval, Québec, G1K 7P4 Canada

**Keywords:** Mesenchymal stem/stromal cells, Dorsal horn neurons, Spinal cord organotypic cultures, Firing activity, Calcium imaging

## Abstract

Conditioned medium obtained from bone marrow-derived stem cells has been proposed as a novel cell-free therapy in spinal cord injury and neuropathic pain, yet the direct effect on spinal neuron function has never been investigated. Here, we adopted spinal cord organotypic cultures (SCOCs) as an experimental model to probe the effect of ST2 murine mesenchymal stem cells-conditioned medium (ST2-CM) on dorsal horn (DH) neuron functional properties. Three days of SCOC exposure to ST2-CM increased neuronal activity measured by Fos expression, as well as spontaneous or induced firing. We showed that the increase in neuronal excitability was associated with changes in both intrinsic membrane properties and an enhanced excitatory drive. The increased excitability at the single-cell level was substantiated at the network level by detecting synchronous bursts of calcium waves across DH neurons. Altogether, SCOCs represent a viable tool to probe mesenchymal cells' effect on intact neuronal networks. Our findings indicate that ST2-CM enhances neuronal activity and synaptic wiring in the spinal dorsal horn. Our data also support the trophic role of mesenchymal cells CM in maintaining network activity in spinal circuits.

**Graphical Abstract:**

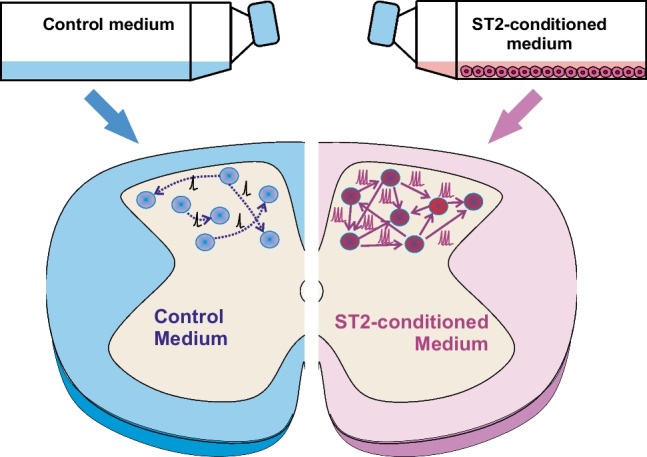

**Supplementary Information:**

The online version contains supplementary material available at 10.1007/s12015-023-10618-x.

## Introduction

Mesenchymal stem cells (MSC) represent a versatile and attractive tool in cell-based therapy for brain repair due to their broad availability in peripheral tissues (e.g. bone marrow or adipose tissue) of adult donors and their capacity to restore homeostatic conditions in different altered areas of the nervous system [1, 2].

In recent years, a growing body of evidence suggests that MSC-derived conditioned media (MSC-CM) have a strong neuroprotective effect in different neurodegenerative diseases, including Parkinson's disease, Alzheimer's disease, and spinal cord injury [3–6]. Neuroprotection is believed to be due to the secretome released by MSC that contains several neuroactive and immunomodulatory molecules promoting neuronal survival, local angiogenesis, and reducing neuroinflammation [7–9]. It remains unclear whether these molecules are freely secreted into the culture medium and/or packed into extracellular vesicles (EV) that act as cargoes for their transport outside the cell [10].

The main advantage of using cell-free conditioned media, as compared to MSC transplantation, is the reduced occurrence of side effects due to the low survival rate of exogenous cells, as well as the immunological responses and rejection mechanisms in host tissues [11]. On the other hand, the safe and rational use of conditioned media in clinical settings requires proper preclinical validation.

*In vivo* models have been largely used in preclinical studies to demonstrate the efficacy and safety of conditioned media in neurological disorders [3, 12–16]; yet, *in vivo* procedures are technically demanding, time-consuming, quite expensive, and require a relatively high number of experimental animals [17].

The organotypic slice cultures of nervous tissue are an *ex vivo* culture model that maintains most of the histological organization and functional properties of the local neural circuitries for several weeks [18]. In particular, the thickness and accessibility of organotypic culture allow for studying the impact of prolonged experimental treatments on both morphological and physiological features of the neuronal networks [19–21]. For these reasons, organotypic cultures represent a convenient link between *in vitro* and *in vivo* models, having a higher level of complexity as compared to cell cultures, but being more permissive than intact animals for analyzing in depth the cellular and molecular profiles of the nervous system, while reducing the number of experimental animals [17, 20].

In this study, we propose to use spinal cord organotypic cultures (SCOCs) from postnatal mice to study the effects of medium conditioned by ST2 murine bone marrow-derived mesenchymal cell line (ST2-CM, [22]). Specifically, we focused on the spinal dorsal horn (DH, [23, 24]), which represents a key central area for the integration of sensory input and a critical site for the development of altered sensory encoding following peripheral nerve and spinal cord injury [25, 26]. We analyzed the impact of ST2-CM on DH network excitability and connectivity by combining functional data from single neurons obtained by patch-clamp recordings with a broader analysis of local neuron activity by calcium imaging.

## Materials and Methods

### Animals

All experimental procedures were approved by the Italian Ministry of Health (authorization 485/2017-PR) and maintained according to the NIH Guide for the Care and Use of Laboratory Animals and to current EU and Italian regulations. Male and female CD1 mice were housed in a controlled and enriched environment and maintained on a 12/12-h light/dark cycle with food and water *ad libitum*.

### ST2 Cell Culture and Conditioned Medium (CM) Preparation

Bone-marrow-derived murine ST2 cells were a kind gift from Professor Rhodri Ceredig (National University of Ireland Galway, Ireland).

Cells were initially cultured in standard growth medium, consisting of Dulbecco's modified Eagle medium/F-12 + GlutaMAX™ (DMEM/F-12), supplemented with 1% penicillin/streptomycin (P/S) and 10% fetal bovine serum (FBS) (all Gibco®, Life Technologies™, Paisley, UK) at 37 °C/5% CO_2_. Cultures reaching 80% confluence were passaged using 0.25% trypsin–EDTA (Gibco®, Life Technologies™).

ST2 cells were seeded into a T75 culture flask at a density of 20,000 cells/cm^2^ in 15 ml DMEM/F-12 and incubated overnight at 37 °C/5% CO_2_ to allow cell adherence. ST2 cells were washed once with phosphate-buffered saline (PBS) and subsequently cultured in 15 ml of Neurobasal medium, composed of Neurobasal A medium) supplemented with 2% 50× B27 supplement (V/V), 1% 200 mM l-glutamine (V/V), and 1% antibiotic/antimycotic (V/V)(all Gibco®, ThermoFisher Scientific, USA) to generate an appropriate conditioned medium CM for subsequent assays on spinal slices. CM was harvested after a further 3 days of incubation, filter sterilized (0.2 μm), and stored at − 80 °C in 1.5 ml aliquots. Control medium samples minus cells were prepared in tandem, following the same protocol.

For a set of experiments, ST2-CM was deprived of EVs [27, 28]. In brief, ST2-CM was centrifuged initially for 20 min at 2,000 g at 4 °C, the supernatant removed and filtered (0.2 μm), before loading onto a 30% sucrose cushion, made using D_2_O (Sigma), and centrifuging at 100,000g for 105 min at 4 °C using an SW28 Ti rotor. The sucrose cushion, washed with at least 1:1 filtered PBS, was then subjected to final centrifugation at 100,000 g for 70 min in a Type 70 Ti fixed angle rotor to pellet EVs, whilst the remaining EV-depleted supernatant was filter sterilized (0.2 μm) and stored at -80 ^o^C.

### SCOC Preparation

**Fig. 1:**
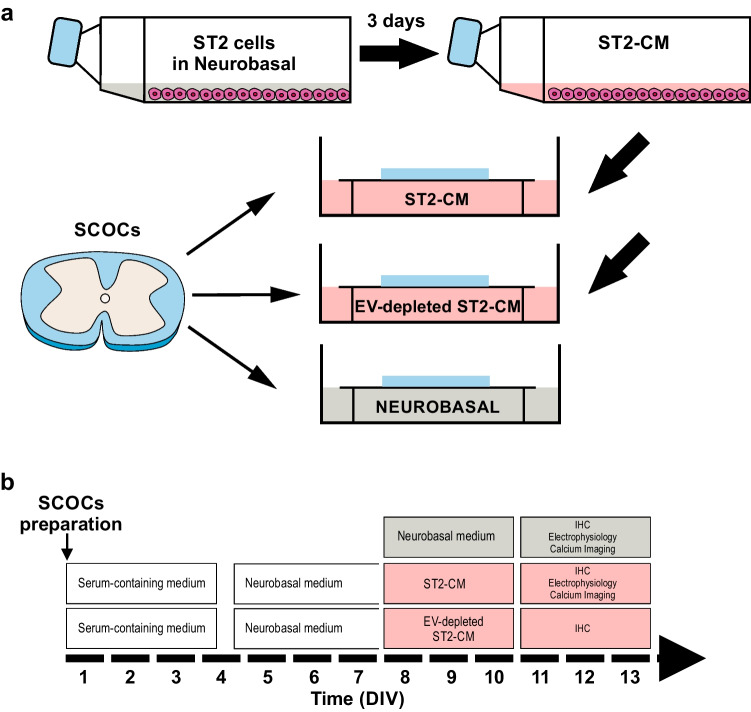
Schematic representation of the experimental design. **a** Diagram illustrating the overall experimental design. CM was obtained following culture and harvest of ST2 cells in neurobasal medium for 3 days. **b** Experimental timeline. SCOCs were exposed to either the neurobasal medium (*grey,* control medium) or ST2-conditioned neurobasal medium (*pink*) before functional/histological experiments. Abbreviations: SCOCs = spinal cord organotypic cultures, ST2 = bone marrow-derived stromal cells, ST2-CM = conditioned medium, EV = extracellular vesicles, DIV = days *in vitro*

SCOCs were prepared as previously described [23, 24]. Briefly, mice were euthanized with a lethal dose of sodium pentobarbital (60 mg/100 g, intraperitoneal). Then, a dorsal laminectomy was performed in an ice-cold cutting solution (containing in mM: 130 N-Methyl-D-glucamine, 10 Glucose, 26 NaHCO_3_, 1.25 NaH_2_PO_4_, 5 MgCl_2_, 0.5 CaCl_2_, 3.5 KCl, and pH adjusted to 7.35). The spinal cord was dissected, the dura mater was gently removed and transverse slices (350 μm thick) were obtained using a vibratome (Leica VT 1200, Germany). The slices were placed on polycarbonate cell culture inserts (Millicell®, Merck, USA) with 0.4 µM pore size. The inserts were then placed in a 35 mm Petri dish containing 1 mL culture medium composed as follows: 50% Eagle’s Basal Medium (V/V), 25% horse serum (V/V), 25% Hanks balanced salt solution (V/V), 0.5% glucose (W/V), 0.5% 200 mM l-glutamine (V/V), and 1% antibiotic/antimycotic (V/V, all Gibco®, ThermoFisher Scientific, USA) and incubated at 34 °C/5% CO_2_. After 3.5 days serum-containing medium was substituted with serum-free Neurobasal medium (ThermoFisher Scientific) supplemented with B27 2% (V/V), L-glutamine 2% (V/V), and antibiotics/antimycotics 1% (V/V) for another 3.5 days. Then, slices were either cultured in Neurobasal™ medium (control condition) or the same medium previously conditioned with bone-marrow-derived murine ST2 cells (ST2-CM) for at least 3 days before the experiment (Fig. 1).

### Patch-Clamp Recordings and Analysis

Recordings were performed as described [23]. SCOCs (DIV > 10) were excised from the inset by cutting the membrane around the slice with a razor blade. The slice and its attached membrane were placed in a recording chamber where were constantly perfused with artificial cerebrospinal fluid (aCSF) at 2 ml/min and bubbled with carbogen (O_2_ 95%/CO_2_ 5%). The aCSF composition was (mM): 126 NaCl, 26 NaHCO_3_, 2.5 KCl, 2 CaCl_2_, 2 MgCl_2_, 10 Glucose, 1.25 NaH_2_PO_4_. The chamber was fixed in a bright field inverted microscope (Eclipse FN-1, Nikon Inc., Japan) equipped with infrared light. Patch-clamp recordings were obtained from visually identified neurons in the spinal dorsal horn by a MultiClamp 700B amplifier (Molecular Devices, USA) and digitized by Digidata 1550b at 20 kHz and filtered at 2.5 kHz. Patch-clamp recordings were obtained in whole-cell configuration (both voltage- and current-clamp modes) and acquired with Clampex software (Molecular Devices). Borosilicate patch pipettes with an average resistance of 5 MΩ were filled with intracellular solution containing (mM): 125 K-gluconate, 20 KCl, 5 EGTA, 2 MgCl_2_, 10 HEPES, 2 ATPNa, 0.2 GTPNa, pH 7.2 (with KOH). All voltage values were corrected offline for liquid junction potential (13.7 mV). Voltage clamp recordings were performed at the holding potential of -70 mV. Current-clamp recordings were obtained either at the resting membrane potential to study spontaneous activity or following the injection of current in 10 pA current-step protocol. The data were analyzed offline with pClamp 10.7.0.3 software (Molecular Devices) for current clamp data or Minianalysis software (Synaptosoft, USA) for voltage clamp data.

### Calcium Imaging Recordings and Analysis

Calcium imaging was performed as previously described [24]. Briefly, SCOCs were loaded with the calcium dye Oregon Green® 488 BAPTA-1, AM (Ex 494, Em 523 nm, ThermoFisher) at 10 µM final concentration with Pluronic F-127 acid 0.04% (W/V), and DMSO at 0.3% (V/V), in 1 ml of Neurobasal medium. Slices were incubated for one hour at 37 °C and 5% CO_2_. Calcium imaging recordings were taken in aCSF bubbled with carbogen by a confocal microscope (Leica, TCS SP5). Images were acquired at 4.9 Hz with a 40× water immersion objective. We selected regions of interest (ROIs) from each digital file with ImageJ software (NIH, USA) for analysis. Changes in fluorescence intensity were plotted in a cartesian chart (intensity, Y-axis; time, X-axis). The fluctuations of fluorescence intensities along the x-axis were analyzed by Clampfit 10.7.0.3 (Molecular Devices) with the “Threshold Search” tool to detect calcium waveforms as positive-ongoing events. The fluorescence bleaching in the file was corrected with Clampfit 10.7.0.3 (Molecular Devices). The threshold for event detection was set to avoid the baseline noise. Bursts were defined as multipeaked waveforms in which one waveform rises in the decay phase of the preceding one.

### Immunohistochemistry

SCOCs were fixed for 1 h at RT in 4% paraformaldehyde (in PB 0.1M, pH 7.4). Following repeated washes with PBS 0.05M, pH 7.4, slices were incubated for 1 h in blocking buffer (PBS containing 1% of normal goat serum and 0.1% Triton-X) at RT. Slices were then incubated overnight at +4 °C with a primary monoclonal antibody raised in rabbits against the Fos protein (1/500; Cell Signaling Technology, cat.# 2250S, USA). Slices were repeatedly washed in PBS and incubated with goat anti-rabbit Alexa-488 secondary antibody (1/500; Invitrogen, USA) in PBS for 3 h at RT. After further washes, slices were mounted using an anti-fade fluorescence-free mounting solution (Sigma). Images were acquired in z-stack (1 µm steps) with a Leica TCS SP5 confocal microscope equipped with a 20× objective. Laser power, gain, and offset were initially set and maintained constant in all the acquisitions. Images were converted to an 8-bit grayscale format and subsequently analyzed with ImageJ software (NIH, Bethesda, Maryland, USA). After delineating the boundaries of the DH laminae according to previous anatomical criteria [29], the immunopositive signal was analyzed by selecting an appropriate threshold. The number of Fos+ cell bodies in the dorsal horn was counted with the “analyze particles” tool of ImageJ and expressed as the number of cells per area (cell density).

### Statistics

Statistical analysis was performed by GraphPad Prism 9 (GraphPad Software, USA). Samples were compared by using an unpaired t-test for independent samples (two-tailed, unless otherwise stated), one-way- or two-way analysis of variance (ANOVA) for interactions between treatment and other variables. All data were reported as mean ± SEM, with n indicating the number of cells, unless otherwise stated. Values of *P* < 0.05 were considered statistically significant.

## Results

### Culturing SCOCs in ST2-CM Increases DH Neuronal Activation

**Fig. 2:**
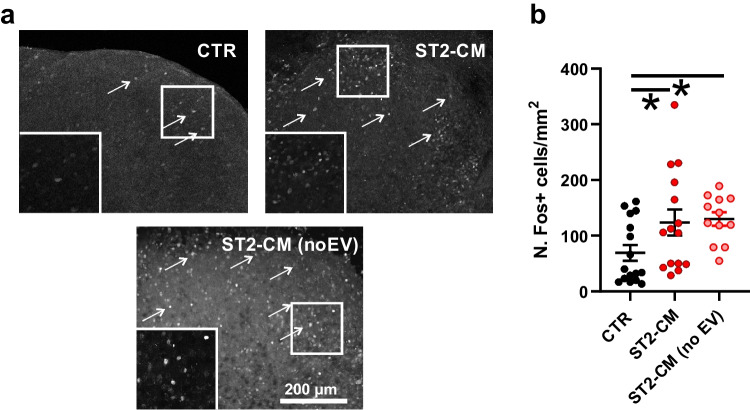
Effect of ST2-CM on Fos expression in the DH of murine SCOCs. **a** representative images of the DH of the spinal cord (13 DIV) under control condition (CTR) or after exposure to ST2-CM for 72 h. The arrows indicate some immunopositive cells. The insets below the images show an enlargement of the DH. **b** Density of Fos-positive nuclei in the mouse DH under control condition (CTR, *n* = 16 DHs) or after exposure to ST2-CM (*n* = 15 DHs; one-way ANOVA with Holm-Šidák post-hoc test, *P* = 0.04) and ST2-CM deprived of EVs (*n* = 12 DHs; *P* = 0.04). Abbreviations: CTR = control, ST2-CM = conditioned medium from bone marrow-derived stromal cells ST2, EV = extracellular vesicles

Murine SCOCs were exposed either to the control medium (Neurobasal) or the ST2 cell conditioned medium (ST2-CM) from DIV 8 to 11 before the experimental procedures (see Fig. 1). In the DH, ST2-CM significantly increased the density of cells expressing Fos, a well-established marker of neuronal activation [30, 31] (Fig. 2a–b), which suggested an increased neuronal activity. A similar increase was also induced by EV-deprived ST2-CM, which suggests that neuronal activation was triggered by factors dissolved into the soluble fraction of the medium.

### ST2-CM Increases Intrinsic DH Excitability

**Fig. 3:**
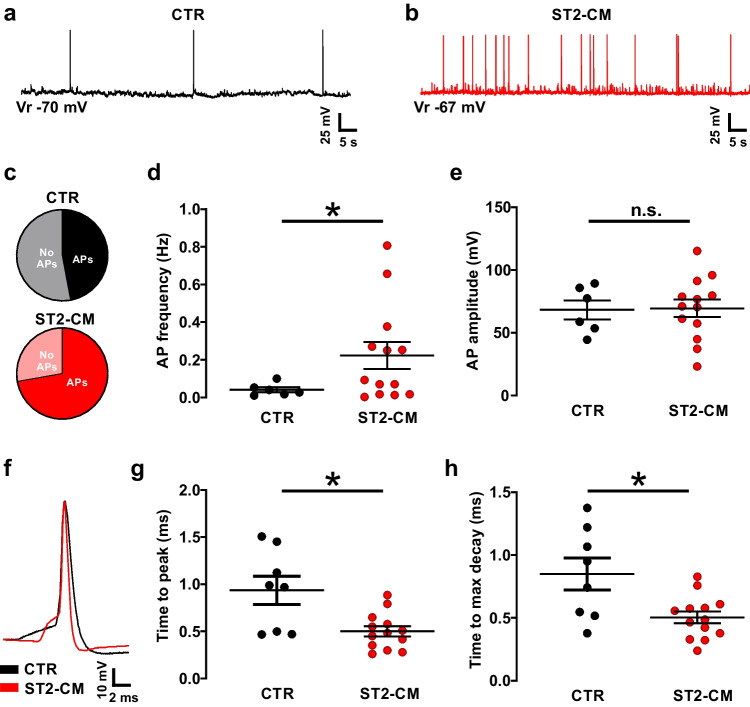
Effect of ST2-CM on the spontaneous firing activity of SCOC DH neurons. **a**–**b** representative traces obtained in current clamp at the resting membrane potential under control conditions (CTR, *black*) or after exposure to ST2-CM (*red*). **c** Pie charts displaying the proportion of neurons with spontaneous APs at rest (47%) in control (*black*) and after exposure to ST2-CM (72%, *red*). **d** Spontaneous AP frequency in control (*black*, *n* = 6) and after exposure to ST2-CM (*red*, *n* = 13; unpaired t-test, *P* = 0.027). **e** Spontaneous AP amplitude in control (*black*, *n* = 6) and after exposure to ST2-CM (*red*, *n* = 13; unpaired t-test, *P* = 0.46). **f** Superimposed APs from control and ST2-CM treated neurons highlighting differences in kinetics. **g** Time to peak of APs from control neurons (*black*, *n* = 8) and ST2-CM treated neurons (*red*, *n* = 13; unpaired t-test, *P* = 0.46). Abbreviations: CTR = control, ST2-CM = conditioned medium from bone marrow-derived stromal cells ST2, AP = action potential

**Fig. 4:**
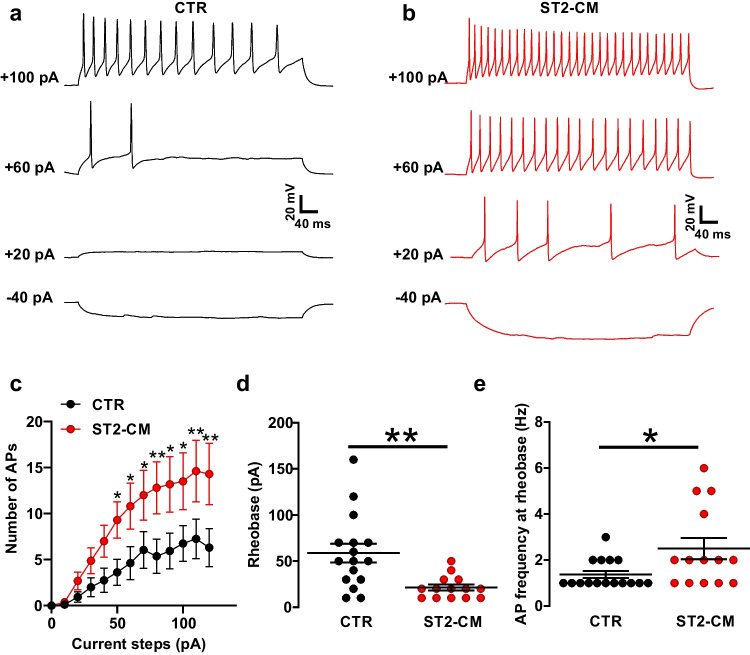
Effect of ST2-CM on the induced firing activity of DH neurons. **a**–**b** representative current clamp recordings obtained in response to injections of depolarizing currents (10 pA per step) in control condition (a, *black*) or after exposure to ST2-CM (b, *red*). **c** Histogram showing the progressive increase in AP numbers along with the increase of depolarizing step in CTR (*black*, *n* = 16) and ST2-CM (*red*, *n* = 16). Note the bigger increment in ST2-CM neurons (two-way ANOVA, interaction between current step and treatment, *P* = 0.002, F = 3.176. Individual comparisons by unpaired t-test, *P < 0.05). **d** Rheobase in control (*black*, *n* = 16) and after exposure to ST2-CM (*red*, *n* = 14; unpaired t-test, *P* = 0.003). **e** AP frequency at rheobase in control (*black*, *n* = 16) and after exposure to ST2-CM (*red*, *n* = 14; unpaired t-test, *P* = 0.02). Abbreviations: CTR = control, ST2-CM = conditioned medium from bone marrow-derived stromal cells ST2, AP = action potential

To address the impact of ST2-CM on neuronal excitability we recorded the spontaneous firing activity of SCOC DH neurons at rest in the current clamp configuration (Fig. 3a–b). While only half of the control neurons exhibited spontaneous action potentials (APs—13 out of 18), firing was observed in about two-thirds of the neurons exposed to ST2-CM (8 out of 17; Fig. 3c). The spontaneous firing frequency was higher in ST2-CM treated neurons (Fig. 3d), while the AP amplitude was unchanged (Fig. 3e). A slight change in AP kinetics was also observed (Fig. 3f), as APs from ST2-CM treated neurons displayed faster rise (Fig. 3g) and decay time (Fig. 3h). Subsequently, we applied a current step protocol to evaluate the impact of ST2-CM on the input–output properties of DH neurons (Fig. 4a–b). The obtained firing patterns (suppl. Fig. 1) corresponded to that described in our previous study on organotypic cultures [23], thus reflecting the heteoregeneity of neuronal cell types in the DH. In both control and ST2-CM treated neurons, we observed a progressive increase in the evoked firing rate along with the increase of the depolarizing step size. However, while in control, the increase in AP frequency induced by the depolarizing steps reached a plateau around 70 pA, after which a further increase in depolarization failed to increase the firing rate (Fig. 4c), in ST2-CM treated cells the number of APs kept growing until the 100 pA (Fig. 4c). Moreover, the rheobase calculated from the current step protocol was lower in ST2-CM treated neurons as compared to control neurons (Fig. 4d) and the AP frequency at the rheobase was significantly higher (Fig. 4e).

**Fig. 5:**
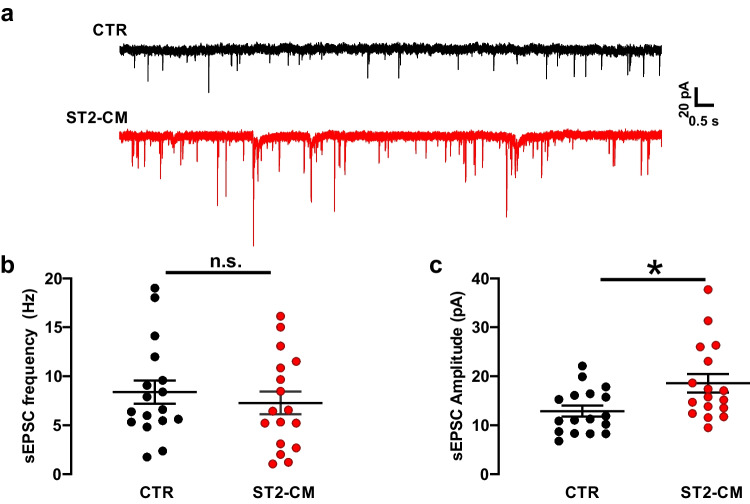
Effect of ST2-CM on the spontaneous excitatory synaptic activity of DH neurons. **a** representative voltage clamp recordings of EPSCs obtained at -70 mV under control conditions (*black*) or after exposure to ST2-CM (*red*). **b** Histogram showing sEPSC frequency in CTR (*black*, *n* = 17) and ST2-CM (*red*, *n* = 17; unpaired t-test, *P* = 0.51). **c** Histogram showing sEPSC amplitude in CTR (*black*, *n* = 17) and ST2-CM (*red*, *n* = 17; unpaired t-test, *P* = 0.02). Abbreviations: CTR = control, ST2-CM = conditioned medium from bone marrow-derived stromal cells ST2, sEPSC = spontaneous excitatory post-synaptic current

The increased excitability in SCOCs could be due to a change in the intrinsic membrane properties of the neurons, but could also account for an increased excitatory input. To address this latter point, we recorded spontaneous excitatory post-synaptic currents (sEPSCs) in voltage clamp at -70 mV (Fig. 5a–b). Interestingly, the frequency of sEPSCs was not affected by ST2-CM (Fig. 5c). Yet, the sEPSC amplitude was increased in ST2-CM treated cells, suggesting an increased excitatory drive (Fig. 5d).

Altogether, these data indicate that DH neurons exposed to ST2-CM displayed increased excitability, which was largely due to altered intrinsic membrane properties. The increased amplitude in sEPSCs can also be explained in terms of postsynaptic alterations (i.e., in the kinetics of the glutamate receptors) and/or a consequence of a higher-level of synchronization in the neuronal network (i.e., leading to a more synchronous release of glutamate).

### ST2-CM Treatment Potentiates Calcium Transients in the DH of SCOCs

**Fig. 6:**
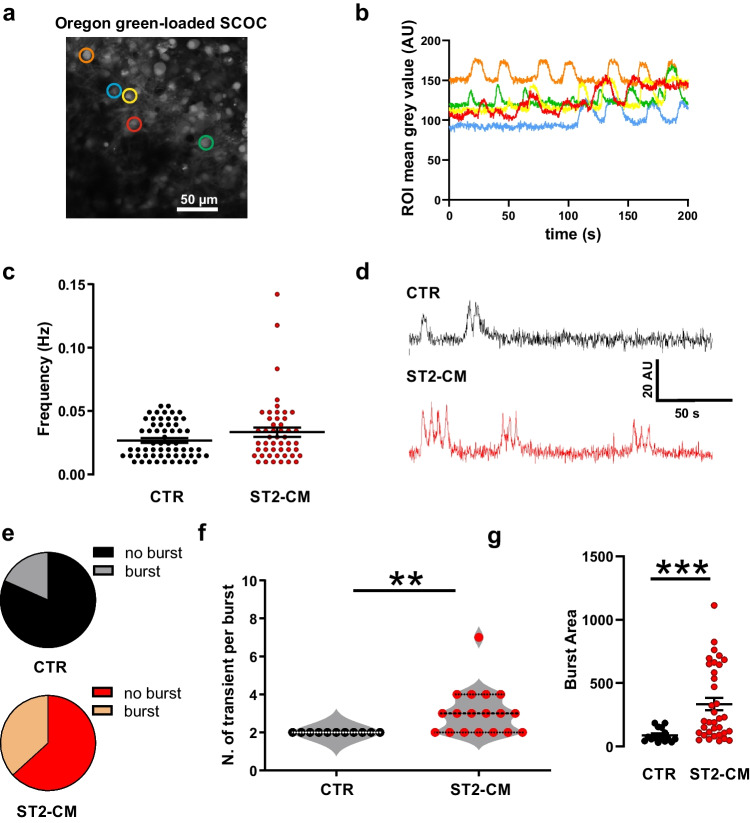
Effect of ST2-CM on calcium transients of DH neurons. **a** A representative image of Oregon green-loaded DH neurons in SCOCs. The colored circles are the ROIs drawn around single DH neurons. **b** changes in mean fluorescence intensity over time per each ROI drawn in a. **c** Calcium transient frequency under control condition (*black*) or after exposure to ST2-CM (*red;* unpaired t-test, *P* = 0.091). **d** Examples of burst activity of calcium transients in a control (*black*, *n* = 60) or after exposure to ST2-CM (*red*, *n* = 49). Note the multipeaked burst waveform in ST2-CM treated neurons. **e** Pie charts displaying the proportion of neurons displaying burst activity in control (18%, *black*) and after exposure to ST2-CM (37%, *red*). **f** Mean number transients per burst per cell in CTR (*black*, *n* = 11) and ST2-CM (*red*, *n* = 18; unpaired t-test, *P* = 0.002). **g** Area under the curve of calcium bursts in CTR (*black*, *n* = 18 bursts) and ST2-CM (*red*, *n* = 35 bursts; unpaired t-test, *P* < 0.001). Abbreviations: CTR = control, ST2-CM = conditioned medium from bone marrow-derived stromal cells ST2, ROI = region of interest, AU = arbitrary unit

While single-cell recordings provide a high-resolution tool to investigate neuronal excitability, the approach does not allow to probe multiple neurons from the same network simultaneously. To address this point, we decided to image neuronal activity in the DH by calcium imaging (Fig. 6a). Recordings from multiple ROIs drawn around single cells show spontaneous calcium transients that often appear in synchronized patterns (Fig. 6b). The observed calcium transients are compatible with those described in neurons [32]. The overall transient frequency is not significantly increased following the exposure to ST2-CM, although a tendency can be observed at least in a subpopulation of cells (one-tailed unpaired t-test, *P* = 0.046; Fig. 6c). However, we noticed that the calcium waveforms in ST2-CM treated neuron are often organized in complex bursts (Fig. 6d). Indeed, bursts were detected in 37% of the ST2-CM treated DH neurons, but only in 18% of the control neurons (Fig. 6e). Moreover, bursts in neurons exposed to ST2-CM displayed more peaks than in control neurons (Fig. 6f) and a larger area under the curve (Fig. 6g).

Overall, CM potentiated the calcium transients in the DH of SCOCs by shifting calcium transients from an asynchronous single peak pattern to synchronous multipeaked waveforms, indicating a more robust organization of excitatory synaptic connectivity following ST2-CM medium treatment.

## Discussion

In the present study, we characterized the functional impact of CM from bone marrow-derived mesenchymal ST2 cells on organotypically cultured spinal cord DH neurons. Our findings demonstrate that soluble factors released by ST2 cells in the culturing medium increase neuronal excitability and improve network activity.

CMs represent an attractive cell-free approach to counteract several neurological diseases [6, 33, 34]. In particular, CM from bone marrow-derived MSC improved functional recovery after spinal cord injury in rats [13, 16, 35] and alleviated neuropathic pain symptoms after nerve injury [36, 37].

Overall, our data support the neurotrophic role of CM from bone marrow-derived MSC on cultured spinal DH neurons. We showed that exposure to an ST2-conditioned medium increases the overall activity of DH neurons and their firing rate. Since EVs released by MSC have been described to reduce neuroinflammation *in vivo* [9], alleviate neuropathic and inflammatory pain symptoms [38], and favor neuronal repair after spinal cord injury [39], we tested whether their release in our medium was responsible for the increased spinal neuron activation. However, depleting the medium from EVs did not affect its capacity to increase FOS, thus suggesting that, in our model, neuronal activity is mainly modulated by soluble factors.

Our data are consistent with previous studies showing that MSC-CM increases and restores Fos reactivity in injured central neurons *in vivo* [40]. Similarly, CM derived from umbilical MSC was shown to foster firing activity in genetically altered induced pluripotent stem cell (iPSC)-derived neurons confirming that factors released in the medium may have a positive modulatory effect on neuronal excitability [41]. However, the real functional impact of CM on neuronal activity has been poorly analyzed in previous studies *in vitro*. Besides confirming a general increase in excitability, our study highlights a direct impact of ST2-CM on active membrane properties of DH neurons as indicated by the decrease in rheobase, the positive shift in input/output firing rate relationship, and the faster AP kinetics. Such changes are typically linked to changes in the composition and properties of voltage-gated potassium and sodium channels [42, 43]. In addition, we observed that the amplitude of sEPSCs was also increased (without a concomitant increase in frequency), which indicates an increased strength of glutamatergic transmission possibly due either to postsynaptic changes [44] or presynaptic plasticity [45]. The impact of increased neuronal excitability and excitatory drive on the overall network activity has been further investigated by the imaging of spontaneous calcium transients. Somatic calcium transients represent a good readout of firing activity [46] and, in spinal organotypic slices, they typically occur in synchronous patterns, especially in developing networks or in the presence of trophic factors [47, 48]. ST2-CM potentiated synchronous calcium transients in the DH by shifting the pattern of calcium waveforms from single spikes to multi-peaked bursts of activity. A more robust and synchronized burst activity suggests a better coupling between the pre-synaptic release of transmitters and the post-synaptic firing response. Since the coincidence of pre-and post-synaptic events is crucial for synaptic strength [49], our data support the role of ST2-CM in promoting synaptic connectivity in the CNS network, as previously experimentally observed by reducing synaptic inhibition or increasing excitation [49–51]. MSC-CM has been also shown to modulate axonal outgrowth in primary neuronal cultures [52], which may represent an additional mechanism to explain the increased connectivity in DH networks.

Altogether, the increased firing activity and intrinsic excitability of DH neurons, the enhanced glutamatergic input, and the synchronous calcium waveforms are typical hallmarks of network maturation and increased connectivity, suggesting an ST2-CM-dependent trophic/consolidating effect on the DH circuits [53]. The composition of CM secretome from mesenchymal stem cells has been investigated in previous studies [5, 54, 55]. Nakano et al. [56] reported that rat bone marrow stromal cells release several growth factors, including IGF-1, VEGF, TGFβ-1, and HGF. Similarly, Cantienieux et al. [13] identified in the same type of medium over twenty molecules involved in apoptosis, inflammation, angiogenesis, and neuromodulation, including neurotrophins such as NGF and BDNF. A similar composition has also been observed in human-derived MSC secretomes, either from bone marrow [57] or from dental pulp [58]. CM from mice bone marrow MSC has been less extensively analyzed, yet a recent study using a high-density protein array has identified up to 21 molecules and growth factors, among which HGF and VEGF [36]. Thus, the positive modulatory effect of ST2 cell secretome on DH neurons observed in the current study is likely due to a cocktail of factors, including neurotrophic factors, that strengthen neuronal activity and synaptic wiring in central circuits.

The DH circuits are critical for the correct encoding of sensory input and can undergo dramatic changes following peripheral nerve or spinal cord injury [25, 26]. Recently, it has been demonstrated that CM from mesenchymal stem cells alleviates neuropathic pain symptoms following nerve injury [36, 37] and is neuroprotective after spinal injury [13]. These important preclinical findings suggest that CM may represent a viable cell-free approach for the treatment of neurological diseases affecting the spinal neurons. Our data support this concept and provide for the first time direct evidence that CM from MSC acts as a positive neuromodulator in central circuits and promotes neuronal activity and synaptic connectivity in DH circuits. A limitation of this study is that the observed effect was not associated with a specific cell population in the DH, thus making more arbitrary any speculation on the direct functional impact on the sensory system, which can be however inferred from the above-cited investigations *in vivo*. On the other hand, being organotypic cultures a model in which a segment of the nervous system has been separated from its peripheral and central connections, the trophic effect exerted by the CM in stimulating neuronal activity and connectivity may explain how factors released by MSC may restore altered circuits in pathological conditions, such as nerve or spinal cord injury, in which proper neuronal connections are lost. Adopting organotypic cultures of central neurons for testing the efficacy of stem cell secretome represents an innovative strategy that may shorten the distance between preclinical studies and therapeutic application.

### Supplementary Information

Below is the link to the electronic supplementary material.

Supplementary file1 (PDF 1426 KB) Supplementary Figure 1. Representative firing patterns are obtained in response to injections of depolarizing currents. a-b. Tonic firing neurons. c. Delayed firing neuron. e. Phasic firing neuron. f. Single (or initial) firing neuron

## Data Availability

The datasets supporting the findings of this study are available upon reasonable request.
