# Supplementary figures and images for: ST2-Conditioned Medium Fosters Dorsal Horn Cell Excitability and Synaptic Transmission in Cultured Mouse Spinal Cord
Source: Stem Cell Rev Rep. 2023 Sep 6;19(8):2918–28. doi: 10.1007/s12015-023-10618-x (PMC10661801; doi:10.1007/s12015-023-10618-x)

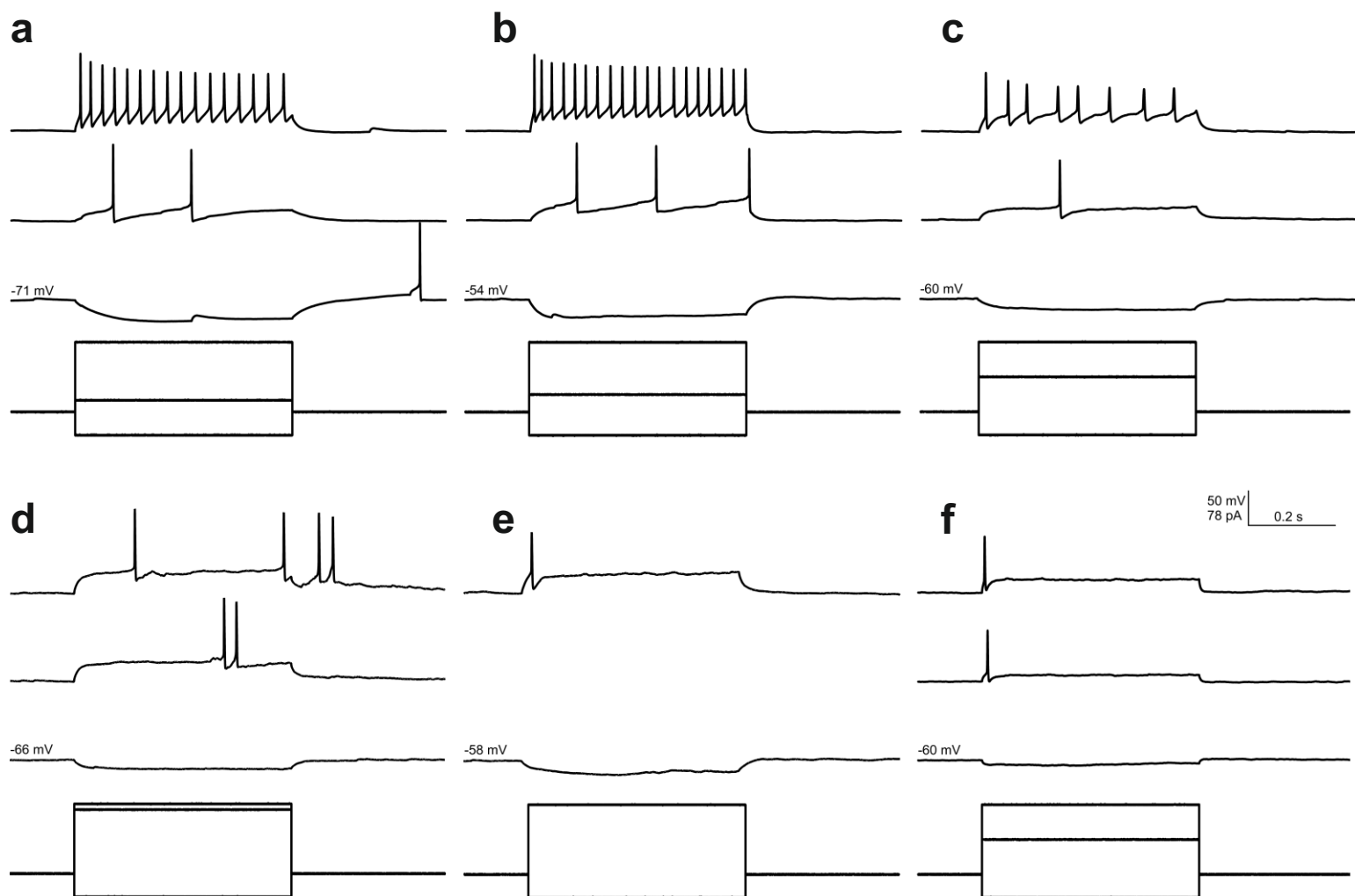

Supplementary Figure 1

Supplement: Supplementary file 1 — Supplementary file1 (PDF 1426 KB) Supplementary Figure 1. Representative firing patterns are obtained in response to injections of depolarizing currents. a-b. Tonic firing neurons. c. Delayed firing neuron. e. Phasic firing neuron. f. Single (or initial) firing neuron [file 12015_2023_10618_MOESM1_ESM.pdf]
